# Toll-like receptor agonists *Porphyromonas gingivalis* LPS and CpG differentially regulate IL-10 competency and frequencies of mouse B10 cells

**DOI:** 10.1590/1678-77572016-0277

**Published:** 2017

**Authors:** Zhiqiang LIU, Yang HU, Pei YU, Mei LIN, Grace HUANG, Toshihisa KAWAI, Martin TAUBMAN, Zuomin WANG, HAN Xiaozhe

**Affiliations:** 1 The Forsyth Institute, Department of Immunology and Infectious Diseases, Cambridge, Massachusetts, United States.; 2 Capital Medical University, Beijing ChaoYang Hospital, Department of Stomatology, Beijing, China.; 3 Sichuan University, West China School of Stomatology, State Key Laboratory of Oral Diseases, Chengdu, Sichuan, China.

**Keywords:** IL-10, *Porphyromonas gingivalis* LPS

## Abstract

IL-10 expressing regulatory B cells (B10) play a key role in immune system balance by limiting excessive inflammatory responses. Effects of toll-like receptor signaling and co-stimulatory molecules on B10 activity during innate and adaptive immune responses are not fully understood.

**Objective:**

This study is to determine the effects of *P. gingivalis* LPS and CpG on B10 cell expansion and IL-10 competency *in vitro*.

**Material and Methods:**

Spleen B cells were isolated from C57BL/6J mice with or without formalin-fixed *P. gingivalis* immunization. B cells were cultured for 48 hours under the following conditions: CD40L, CD40L+LPS, CD40L+CpG, and CD40L+LPS+CpG in the presence or absence of fixed *P. gingivalis*. Percentages of CD1d^hi^CD5^+^ B cells were measured by flow cytometry. IL-10 mRNA expression and secreted IL-10 were measured by real-time quantitative PCR and by ELISA respectively.

**Results:**

*P. gingivalis* LPS plus CD40L significantly increased CD1dhiCD5+ B cell percentages and secreted IL-10 levels in both immunized and non-immunized mice B cells in the presence or absence of *P. gingivalis*, compared with control group. Secreted IL-10 levels were significantly increased in CD40L+LPS treated group compared with CD40L treatment group in the absence of *P. gingivalis*. CpG plus CD40L significantly decreased CD1d^hi^CD5^+^ B cell percentages, but greatly elevated secreted IL-10 levels in immunized and non-immunized mice B cells in the absence of *P. gingivalis*, compared with CD40L treatment group.

**Conclusions:**

*P. gingivalis* LPS and CpG differentially enhance IL-10 secretion and expansion of mouse B10 cells during innate and adaptive immune responses.

## Introduction

IL-10 expressing regulatory B cells (B10) is a specific IL-10 competent regulatory B cell subset that has been recently identified in both mice and humans^27^. B10 cell down-regulates autoimmune disease, inflammation and immune responses through IL-10 expression, playing crucial regulatory roles in innate and adaptive immunity^27^. Though mouse B10 cells share some overlapping phenotypic markers with other multiple phenotypically defined B cell subsets, they have been found to be predominantly enriched in spleen CD1d^high^CD5^+^ B cells^27^.

Toll-like receptors (TLRs), which belong to pattern recognition receptors, are specialized transmembrane proteins that mediate innate immunity through detecting common structures of many microbial species such as bacterial lipopolysaccharides (LPS) or viral nucleic acids^17,25^. Upon recognition of a pathogen, TLRs initiate a signaling cascade that leads to expression and release of pro-inflammatory cytokines, chemokines, and Type-I interferons^8,21^. *Porphyromonas gingivalis* (*P. gingivalis*) LPS has been shown to be able to activate both TLR2 and TLR4 due to its unique structure and function^2,6^, and CpG is known as TLR9 agonist to stimulate the immune responses^9,16^.

Interaction between CD40 Ligand (CD40L) and CD40 plays an important role in the initiation and progression of cellular and humoral adaptive immunity^15^. The activation of CD40 on B cells by CD40L is crucial for T cell-dependent B cell proliferation, differentiation, and antibody isotype switching^11,13,14^.

Recent studies demonstrated that culturing spleen B cells with LPS or CD40L for 48 h induced significantly higher frequencies of cytoplasmic IL-10 production in B cells than control *in vitro* ^20^. LPS stimulation of spleen B cells for 24 h induced more IL-10 than unstimulated cells^28^. Spleen B cells with a CD1d^high^CD21^+^CD23^-^ MZ phenotype can produce IL-10 in response to CpG stimulation in mice with lupus-like autoimmune disease^30^. However, despite all these findings, the effects of TLR agonists along with co-stimulatory molecules, such as CD40L, on B10 activity during innate and adaptive immune responses are not clearly understood. Furthermore, there is limited information on the role of B10 cells during immune responses to oral diseases, such as periodontal disease, when encountering oral pathogens and their derivatives. In the present study, spleen B cells from *P. gingivalis* non-immunized and immunized mice were co-stimulated with TLR4, TLR9, and CD40 signals to investigate their effects on B10 cell expansion and IL-10 competency *in vitro*.

## Material and Methods

### P. gingivalis culture and fixation

*P. gingivalis* (strain ATCC 33277) were grown on anaerobic blood agar plates (NHK agar, Northeast Laboratory, Waterville, ME, U.S.A.) in an anaerobic chamber with 85% N_2_, 5% H_2_, and 10% CO_2_. Single colony of *P. gingivalis* was isolated from the plate and grown in ATCC Medium 2722. After incubation at 37°C for 4 d, bacteria number in culture medium was determined by reading optical density values using spectrophotometer and comparing them with a curve derived from a standard plate count. Bacteria were collected and fixed with 4% paraformaldehyde (PFA) for 30 min at room temperature, then washed three times with sterile phosphate-buffered saline (PBS) and resuspended in PBS at the concentration of 5×10^8^/mL.

### Animals

C57BL/6J mice (Jackson Laboratory, Bar Harbor, ME, U.S.A.) aging 8-10 weeks were equally and randomly divided into four groups. Group 1 and 2 were set as non-immunized mice groups in which mice were sacrificed directly for spleen B cell isolation. Group 3 and 4 were set as immunized mice groups and mice were immunized by 1×10^8^ fixed *P. gingivalis* intraperitoneal injection at day 0, then followed by 1×10^7^ fixed *P. gingivalis* injection at day 7 to enhance the immunization. Mice were sacrificed for B cell isolation at day 10. All mice used in the study were maintained under pathogen-free conditions in laminar flow cabinets. Experimental protocols were approved by the Institutional Animal Care and Use Committee of the Forsyth Institute.

### B cell isolation

Mice were euthanized in CO_2_ chamber and spleens were harvested. Single splenic cells were yielded by grinded on a steel mesh and then filtered with 100 μm Cell Strainers. After red blood cells removal by Ammonium-Chloride-Potassium (ACK) lysis buffer (Life Technologies, Carlsbad, CA, USA), splenic cells were resuspended in PBS and filtered with 40 μm Cell Strainers. Then non-B cells were magnetically labeled using Pan B cell isolation kit (Miltenyi Biotec, Cambridge, MA, USA). Briefly, single splenic cell suspensions were incubated with biotin-conjugated monoclonal antibodies against non-B cell surface markers (CD4, CD11c, CD49b, CD90, Gr-1, and Ter119) at 4°C for 10 min followed by incubation with magnetic microbeads conjugated anti-biotin antibodies at 4°C for 15 min. Magnetically labeled cells were then depleted by passing through LD columns (Miltenyi Biotec, Cambridge, MA, USA) under the magnetic field of the QuadroMACS™ Separator (Miltenyi Biotec, Cambridge, MA, USA). Unlabeled cells that passed through LD column were collected (contained >98.5% CD19+ cells).

### B cell culture

B cell number was counted by hemacytometer. Each 1×10^6^ B cells were cultured in 200 μL IMDM+GlutaMAX^TM^ (Life Technologies, Carlsbad, CA, USA) complete medium (contains 10% FCS, 100 U/mL penicillin, 100 mg/mL streptomycin, 2 mM L-glutamine, 2.5 μg/mL Amphotericin B and 50 μM 2-ME) in 96-well plates under the following conditions: control, CD40L, CD40L+LPS, CD40L+CpG, or CD40L+LPS+CpG in the absence or in the presence of fixed *P. gingivalis*. Final concentrations of these stimulants were as follows: CD40L (eBioscience, San Diego, CA, USA, 1 μg/mL), *P. gingivalis* LPS (Invivogen, San Diego, CA, USA, 10 μg/mL), mouse CpG-DNA (Hycult, Plymouth Meeting, PA, USA, 10 μM), and fixed *P. gingivalis* (5×10^6^/1×10^6^ cells). *P. gingivalis* LPS was used as TLR4 agonist and mouse CpG-DNA(5’-TCCATGACGTTCCTGATGCT -3’) was used as TLR9 agonist. B cells cultured without stimulation were used as control. Cells were cultured in a humidified incubator at 37°C with 5% CO_2_ for 48 h. Thirty of 200 μL medium in each well was used to determine CD1d^high^CD5^+^ B cell percentages and remaining cells were used to determine IL-10 mRNA expression levels. Culture supernatant was used for secreted IL-10 levels measurement.

### CD1dhighCD5+ B cells percentages determination

B cells were washed with PBS and Fc receptors were blocked by incubating with TruStain fcX^TM^ (BioLegend, San Diego, CA, USA) on ice for 10 min, then followed by incubation with PE anti-mouse CD1d (BioLegend, San Diego, CA, USA) and Alexa Fluor 647 anti-mouse CD5 fluorescence conjugated antibodies (BioLegend, San Diego, CA, USA) on ice for 30 m using predetermined optimal concentrations. Then, all cells were counted by flow cytometers (BD Biosciences, San Jose, CA, USA) and data were analyzed by FlowJo v10 software. For each sample, the same gate was applied to the other samples to determine their CD1d^high^CD5^+^ B cell percentages. Since mouse B10 cells has been found to be predominantly enriched in spleen CD1d^high^CD5^+^ B cells, CD1d^high^CD5^+^ B cell percentage is considered as the proportional indicator of B10 cell percentage.

### IL-10 mRNA expression measurement

Total mRNA of B cells was isolated by PureLink RNA Mini Kit (Life Technologies, Carlsbad, CA, USA) following the manufacturer’s instructions. Isolated mRNA was then reverse transcribed to cDNA using the SuperScript™ II Reverse Transcriptase system (Invitrogen, San Diego, CA, USA) in the presence of random primers following the manufacturer’s instructions. Then, real-time quantitative PCR (RT-qPCR) was carried out in a 20 μL reaction system using LightCycler 480 SYBR Green I Master kit (Roche Diagnostics, Indianapolis, IN, USA) and LightCycler 480 Instrument (Roche Diagnostics, Indianapolis, IN, USA). 2 μL cDNA template was used for each sample and measured in duplicate. 250 nM pre-designed IL-10 (Invitrogen, San Diego, CA, USA) or GAPDH primers (Sigma, St. Louis, MO, USA) were used and their sequences were as follows: IL-10, forward 5’-GACCAGCTGGACAACATACTGCTAA-3’ and reverse 5’-GATAAGGCTTGGCAACCCAAGTAA-3’; GAPDH, forward 5’-CCCCAGCAAGGACACTGAGCAA-3’ and reverse 5’- GTGGGTGCAGCGAACTTTATTGATG-3’. RT-qPCR conditions were: 95°C for 10 min, followed by 45 cycles of 95°C for 10 s, 55°C for 15 s, and 72°C for 30 s. Melting curves were acquired to check the target cDNA amplification specificity. IL-10 mRNA expression level were presented as fold changes relative to GAPDH reference.

### Secreted IL-10 level measurement

Secreted IL-10 levels in the cultured supernatant were measured by Mouse IL-10 ELISA MAX Standard Kit (BioLegend, San Diego, CA, USA) following the manufacturer’s manual. All samples were immediately 1:1 diluted prior to measure and measured in duplicate. Absorbance values were read by Synergy HT Microplate Reader (Biotek, Winooski, VT, USA) at 450 nm and IL-10 concentrations were calculated according to standard curve and dilution ratio.

### Statistical analysis

All quantitative data were expressed as means±SD. Statistical analysis was performed using Student’s t*-*test for comparisons of two groups. Statistical significance was set at p<0.05.

## Results

### Non-immunized mice CD1dhighCD5+ B cell expansion with CD40L, LPS, and CpG treatment with/without P. gingivalis co-stimulation

**Figure 1:**
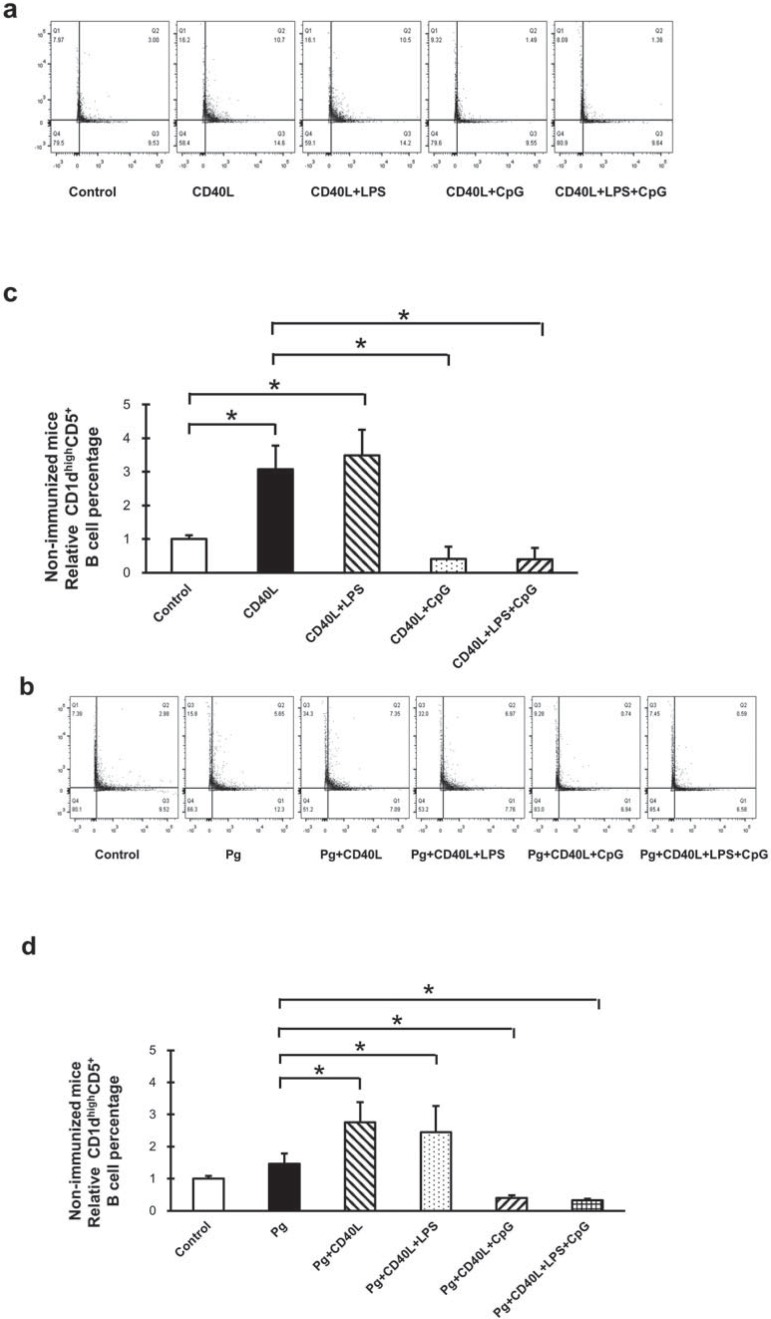
B10 cell expansion in non-immunized mouse splenocyte B cells after CD40L, LPS, and CpG treatment with/without *P. gingivalis* co-stimulation. Splenocyte B cells were separated from non-immunized C57/BL6J mice and cultured 48 hours with CD40L (1 mg/mL), CD40L (1 mg/mL)+*P. gingivalis* LPS (10 mg/mL), CD40L(1 mg/mL)+CpG (10 mM), and CD40L (1 mg/mL)+*P. gingivalis* LPS (10 mg/mL)+CpG (10 mM) in the absence or in the presence of fixed *P. gingivalis* (5×106*per* 1×106 cells). CD1highCD5+ B cells were detected using flow cytometry in control and treatment groups without *P. gingivalis* (a) and with *P. gingivalis* (b) (X-axis: CD5 PE staining; Y-axis: CD1d APC staining). The percentage of CD1highCD5+ B cells was quantified and analyzed by FlowJo software in control and treatment groups without *P. gingivalis* (c) and with *P. gingivalis* (d) (mean±SD, n=3, *p<0.05, detailed statistics information in Table 1)

B cells separated from non-immunized mice splenocytes were cultured for 48 h under multiple conditions including CD40L, CD40L+LPS, CD40L+CpG, and CD40L+LPS+CpG (Figure 1a); *P. gingivalis*, *P. gingivalis*+CD40L, *P. gingivalis*+CD40L+LPS, *P.g*+CD40L+CpG, and *P. gingivalis*+CD40L+LPS+CpG (Figure 1b). The percentage of CD1d^high^CD5^+^ B cell was measured and quantified by flow cytometry for each group. Compared with non-treatment control group, CD40L significantly induced CD1d^high^CD5^+^ B cell expansion and CD40L+LPS had similar significant induction; however, additional CpG significantly suppressed CD40L-induced CD1d^high^CD5^+^ B cell expansion with or without LPS (Figure 1c). CD1d^high^CD5^+^ B cell population had no significant change under fixed *P. gingivalis* treatment only, comparing with non-treatment control group; and the induction by CD40L and CD40L+LPS or suppression by additional CpG were not affected by additional *P. gingivalis* treatment (Figure 1d). Taken together, with or without *P. gingivalis* treatment on non-immunized mice splenocyte B cells, CD40L and CD40L+LPS significantly induced CD1d^high^CD5^+^ B cell expansion and CpG reduced this expansion significantly (Table 1).

**Table 1:** Analysis of CD1dhighCD5+ B cells percentage statistics in groups without *P. gingivalis* treatment

Fig.1c	Control	CD40L	CD40L+LPS	CD40L+CpG	CD40L+LPS+CpG
Control		p<0.05	p<0.05	p<0.05	p<0.05
CD40L	p<0.05		p>0.05	p<0.05	p<0.05
CD40L+LPS	p<0.05	p>0.05		p<0.05	p<0.05
CD40L+CpG	p<0.05	p<0.05	p<0.05		p>0.05
CD40L+LPS+CpG	p<0.05	p<0.05	p<0.05	p>0.05	

**Fig.2c**	**Control**	**CD40L**	**CD40L+LPS**	**CD40L+CpG**	**CD40L+LPS+CpG**

Control		p<0.05	p<0.05	p<0.05	p<0.05
CD40L	p<0.05		p<0.05	p<0.05	p<0.05
CD40L+LPS	p<0.05	p<0.05		p<0.05	p<0.05
CD40L+CpG	p<0.05	p<0.05	p<0.05		p>0.05
CD40L+LPS+CpG	p<0.05	p<0.05	p<0.05	p>0.05	

### Immunized mice CD1dhighCD5+ B cell expansion with CD40L, LPS, and CpG treatment with/without P. gingivalis co-stimulation

**Figure 2:**
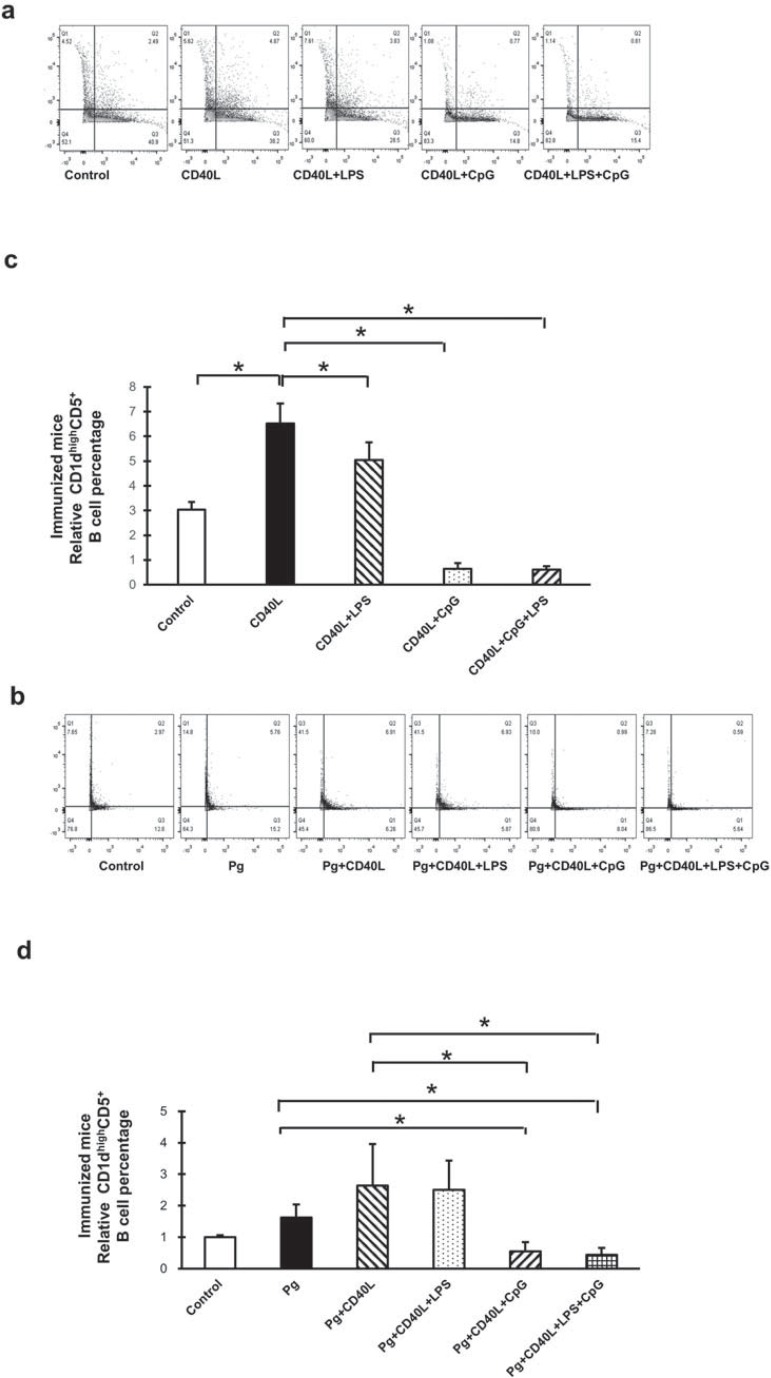
B10 cell expansion in immunized mouse splenocyte B cells after CD40L, LPS, and CpG treatment with/without *P. gingivalis* co-stimulation. C57BL/6J mice were immunized by intraperitoneal injection of fixed *P. gingivalis* on day 0 (1×106) and day 7 (1×105). Splenocyte B cells were separated from immunized mice on day 10 and cultured 48 hours with CD40L (1 mg/mL), CD40L (1 mg/mL)+*P. gingivalis* LPS (10 mg/mL), CD40L(1 mg/mL)+CpG (10 mM), and CD40L (1 mg/mL)+*P. gingivalis* LPS (10 mg/mL)+CpG (10 mM) in the absence or in the presence of fixed *P. gingivalis* (5×106*per* 1×106 cells). CD1highCD5+ B cells were detected using flow cytometry in control and treatment groups without *P. gingivalis* (a) and with *P. gingivalis* (b) (X-axis: CD5 PE staining; Y-axis: CD1d APC staining). The percentage of CD1highCD5+ B cells was quantified and analyzed by FlowJo software in control and treatment groups without *P. gingivalis* (c) and with *P. gingivalis* (d) (mean±SD, n=3, *p<0.05, detailed statistics information in Table 2)

B cells separated from immunized mice splenocytes were cultured for 48 h under the same conditions previously mentioned and measured by flow cytometry (Figures 2a, 2b). Compared with non-treatment control group, CD40L significantly induced CD1d^high^CD5^+^ B cell expansion and additional LPS slightly reduced this induction; also, additional CpG largely suppressed CD40L-induced CD1d^high^CD5^+^ B cell expansion with or without LPS (Figure 2c). For immunized mice, CD1d^high^CD5^+^ B cell population also had no significant change with fixed *P. gingivalis* treatment alone, comparing with non-treatment control group; and the induction by CD40L or suppression by additional CpG were not affected by additional *P. gingivalis* treatment. However, additional LPS inhibition effect on CD40L induction was vanished with *P. gingivalis* treatment (Figure 2d) comparing with no *P. gingivalis* treatment (Figure 2c) in immunized mice B cells (Table 2). These results indicated that B cells from *P. gingivalis* immunized mice had partial similar responses for CD40L, CD40L+LPS, and CD40L+CpG treatment with or without *P. gingivalis* co-stimulation, compared with B cells from non-immunized mice. Other than that, LPS significantly reduced the expansion of immunized CD1d^high^CD5^+^ B cell without *P. gingivalis* (Figure 2c), but had no effect on non-immunized CD1d^high^CD5^+^ B cell (Figure 1c); also, compared with *P. gingivalis* only treatment group, CD40L+*P. gingivalis* treatment had no significant induction in immunized CD1d^high^CD5^+^ B cell (Figure 2d), but induced significant expansion in non-immunized CD1d^high^CD5^+^ B cell (Figure 1d).

**Table 2:** Analysis of CD1dhighCD5+ B cells percentage statistics in groups with *P. gingivalis* treatment

Fig.1d	Control	P.g	P.g+CD40L	P.g+CD40L+LPS	P.g+CD40L+CpG	P.g+CD40L+LPS+CpG
Control		p<0.05	p<0.05	p<0.05	p<0.05	p<0.05
P.g	p<0.05		p<0.05	p<0.05	p<0.05	p<0.05
P.g+CD40L	p<0.05	p<0.05		p>0.05	p<0.05	p<0.05
P.g+CD40L+LPS	p<0.05	p<0.05	p>0.05		p<0.05	p<0.05
P.g+CD40L+CpG	p<0.05	p<0.05	p<0.05	p<0.05		p>0.05
P.g+CD40L+LPS+CpG	p<0.05	p<0.05	p<0.05	p<0.05	p>0.05	

**Fig.2d**	**Control**	**P.g**	**P.g+CD40L**	**P.g+CD40L+LPS**	**P.g+CD40L+CpG**	**P.g+CD40L+LPS+CpG**

Control		p<0.05	p<0.05	p<0.05	p<0.05	p<0.05
P.g	p<0.05		p>0.05	p>0.05	p<0.05	p<0.05
P.g+CD40L	p<0.05	p>0.05		p>0.05	p<0.05	p<0.05
P.g+CD40L+LPS	p<0.05	p>0.05	p>0.05		p<0.05	p<0.05
P.g+CD40L+CpG	p<0.05	p<0.05	p<0.05	p<0.05		p>0.05
P.g+CD40L+LPS+CpG	p<0.05	p<0.05	p<0.05	p<0.05	p>0.05	

### IL-10 mRNA levels in B cells from non-immunized and immunized mice with CD40L, LPS, and CpG treatment with/without P. gingivalis co-stimulation

**Figure 3:**
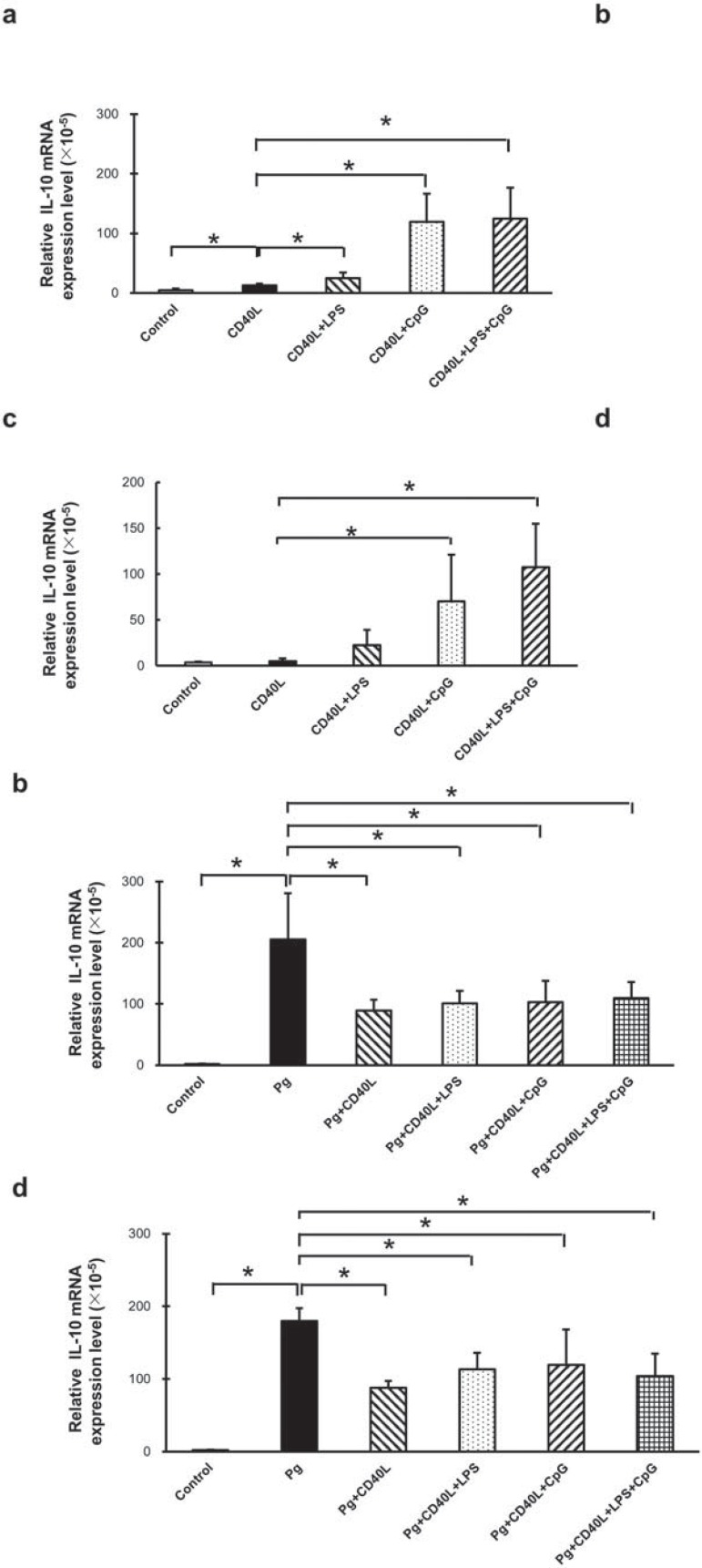
IL-10 mRNA expression in B cells from non-immunized and immunized mice with CD40L, LPS, and CpG treatment with/without *P. gingivalis* co-stimulation. Splenocyte B cells were separated and cultured 48 hours with CD40L (1 mg/mL), CD40L (1 mg/mL)+*P. gingivalis* LPS (10 mg/mL), CD40L (1 mg/mL)+CpG (10 mM), and CD40L (1 mg/mL)+*P. gingivalis* LPS (10 mg/mL)+CpG (10 mM) in the absence or in the presence of fixed *P. gingivalis* (5×106*per* 1×106 cells). IL-10 expressions were determined by RT-qPCR in control and treatment groups without *P. gingivalis* (a) and with *P. gingivalis* (b) from non-immunized C57/BL6J mice, and same groups from immunized C57/BL6J mice without *P. gingivalis* (c) and with *P. gingivalis* (d) (mean±SD, n=3, *p<0.05)

IL-10 mRNA levels were measured and analyzed by RT-qPCR in cultured B cells separated from non-immunized mice (Figures 3a and 3b) and immunized mice (Figures 3c and 3d) with multiple treatments including CD40L, CD40L+LPS, CD40L+CpG, and CD40L+LPS+CpG (Figures 3a and 3c); *P. gingivalis*, *P. gingivalis*+CD40L, *P. gingivalis*+CD40L+LPS, *P. gingivalis*+CD40L+CpG, and *P. gingivalis*+CD40L+LPS+CpG (Figures 3b and 3d). Comparing with non-treatment control group, CD40L significantly increased IL-10 mRNA expression and additional LPS enhanced this increase in B cells from non-immunized mice (Figure 3a), but not from immunized mice (Figure 3c). However, additional CpG largely increased IL-10 mRNA expression compared with CD40L treatment with or without LPS in B cells from both types of mice (Figures 3a and 3c). *P. gingivalis* stimulation significantly increased IL-10 mRNA expression, and this enhancement was significantly suppressed with additional CD40L, CD40L+LPS, CD40L+CpG, and CD40L+LPS+CpG in B cells from both types of mice (Figures 3b and 3d). Taken together, Cd40L+LPS and CD40L+CpG induced significant increase of IL-10 mRNA expression without *P. gingivalis* treatment; however, these additional combinations suppressed the induction of IL-10 mRNA expression caused by *P. gingivalis*.

### Secreted IL-10 levels in B cells from non-immunized and immunized mice with CD40L, LPS, and CpG treatment with/without P. gingivalis co-stimulation

**Figure 4:**
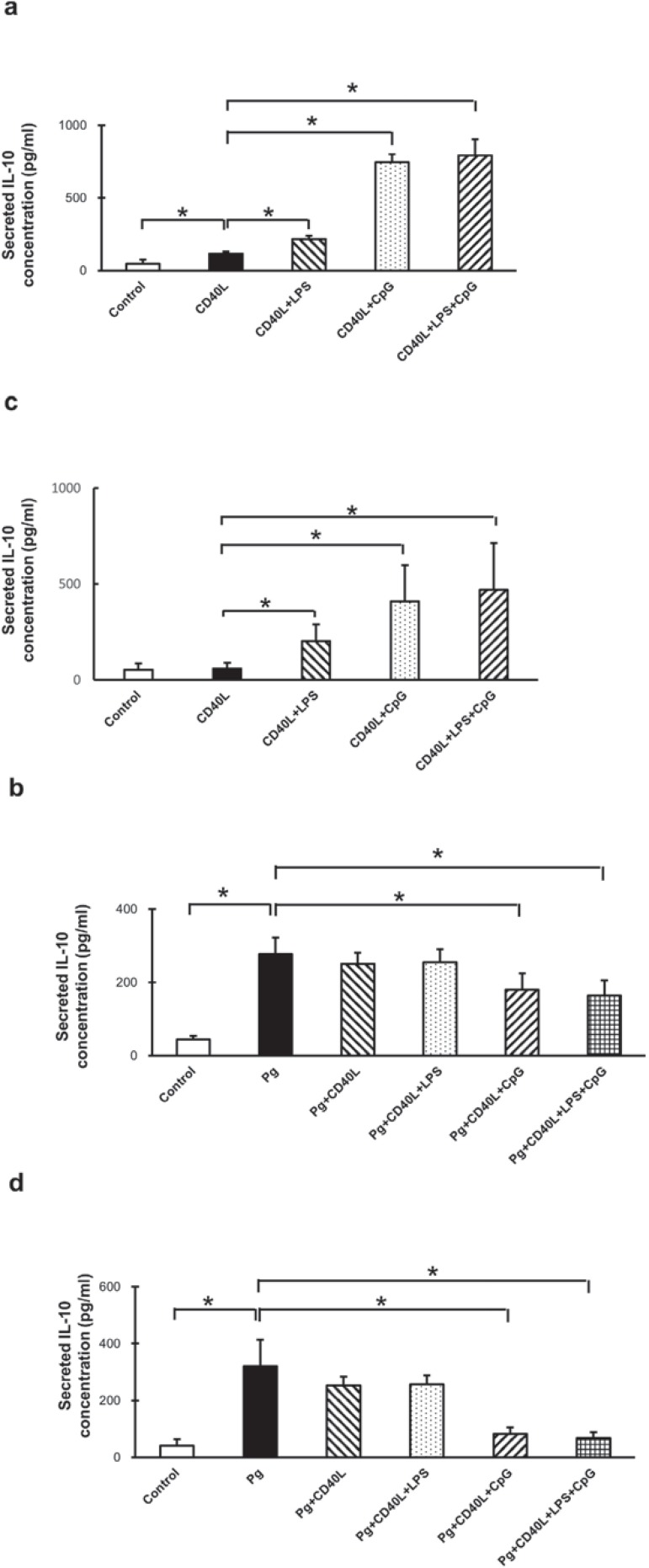
Secreted IL-10 levels in B cells from non-immunized and immunized mice with CD40L, LPS, and CpG treatment with/without *P. gingivalis* co-stimulation. Splenocyte B cells were separated and cultured 48 hours with CD40L (1 mg/mL), CD40L (1 mg/mL)+*P. gingivalis* LPS (10 mg/mL), CD40L (1 mg/mL)+CpG (10 mM), and CD40L (1 mg/mL)+*P. gingivalis* LPS (10 mg/mL)+CpG (10 mM) in the absence or in the presence of fixed *P. gingivalis* (5×106*per* 1×106 cells). Medium supernatants were collected and secreted IL-10 protein levels were measured by ELISA in control and treatment groups without *P. gingivalis* (a) and with *P. gingivalis* (b) from non-immunized C57/BL6J mice, and same groups from immunized C57/BL6J mice without *P. gingivalis* (c) and with *P. gingivalis* (d) (mean±SD, n=3, *p<0.05)

Secreted IL-10 levels were measured and analyzed by ELISA from supernatant of cultured B cells separated from non-immunized mice (Figure 4a and 4b) and immunized mice (Figures 4c and 4d). Comparing with non-treatment control group, CD40L significantly increased IL-10 secretion in B cells from non-immunized mice only and additional LPS enhanced this increase in B cells from both type of mice (Figures 4a and 4c). Also, additional CpG largely increased IL-10 secretion compared with CD40L treatment with or without LPS in B cells from both types of mice (Figures 4a and 4c). *P. gingivalis* stimulation alone significantly increased IL-10 secretion and this induction was significantly suppressed with additional CD40L+CpG, but additional CD40L and Cd40L+LPS had no impacts on this induction (Figures 4b, 4d). These results suggested that Cd40L+LPS and CD40L+CpG significantly increased IL-10 secretion without *P. gingivalis* treatment; however, *P. gingivalis* treatment significantly induced IL-10 secretion, additional Cd40L+LPS had no effect, and CD40L+CpG significantly inhibited this induction.

## Discussion

IL-10 producing B10 cells play an essential role in immune system balance by suppressing excessive inflammatory responses^18,20,30^. However, little is known about the effects of co-stimulation by multiple TLR agonists and CD40 activator on B10 cells under different immunological conditions. In the present study, we investigated the changes of B10 cell population and IL-10 secretion by combined treatment of *P. gingivalis* LPS (TLR4 agonist), CpG (TLR9 agonist), and CD40L in the context of innate immunity (cells from non-immunized mice) and adaptive immunity (cells from immunized mice). The results showed that *P. gingivalis* LPS enhanced IL-10 secretion by B10 cells in mice *in vitro* during innate and adaptive immune responses with increased CD1d^high^CD5^+^ B cells; However, CpG was more effective than *P. gingivalis* LPS to enhance IL-10 competency during these responses with decreased CD1d^high^CD5^+^ B cells.

*P. gingivalis* LPS is a purified product of lipopolysaccharide from Gram-negative bacteria *Porphyromonas gingivalis*, which is considered as the main pathogen of periodontal disease^3,12,26^. LPS is the major component of Gram negative bacteria that activates the innate immune system^4,29^. However, *P. gingivalis* LPS has a unique and heterogenous chemical structure, which is different from traditionally recognized enteric bacterium-derived LPS such as *E. coli* LPS^7,22,23^. *P. gingivalis* LPS and *E. coli* LPS have been shown to trigger different intracellular inflammatory signaling pathways^7^ and cytokine productions^1,22^. It was suggested that the structural heterogeneity of *P. gingivalis* lipid A contributes to the unusual innate host response to this LPS and its ability to interact with both TLR2 and TLR4^2,6^. This may explain the difference of the induction effects on B10 population and IL-10 secretion between co-stimulating *P. gingivalis* LPS plus CD40L and *E. coli* LPS plus CD40L in non-immunized mice^19^. In non-immunized mice without *P. gingivalis* treatment, additional *P. gingivalis* LPS did not further increase CD1d^high^CD5^+^ B cells percentages (Figure 1c), but significantly increased IL-10 mRNA expression (Figure 3a) and IL-10 secretion (Figure 4a) compared with CD40L only group. Moreover, in immunized mice without *P. gingivalis* treatment, *P. gingivalis* LPS suppressed the expansion of CD1d^high^CD5^+^ cells induced by CD40L (Figure 2c) with an increase of IL-10 secretion (Figure 4c). These results suggest that *P. gingivalis* LPS stimulation has different effects on innate and adaptive immune responses, and it may enhance the IL-10 secretion from fewer CD1d^high^CD5^+^ B cells with higher competence or from increased B10 cells other than CD1d^high^CD5^+^ cell subset. These differences and possible mechanisms need to be investigated in future studies. *Porphyromonas gingivalis* synthesizes two LPS, O-LPS and A-LPS. The structures of the O-PS and A-PS repeating units, the core oligosaccharide (OS), and the linkage of the repeating unit to the core in O-LPS and A-LPS have been extensively studied^24^. Analysis of the detailed structure of *P. gingivalis* LPS is essential for further mechanistic investigation of the antigenicity of this important periodontal pathogen.

*P. gingivalis* induces periodontitis through the disruption of the host tissue homeostasis and adaptive immune response, which allows uncontrolled growth of the commensal microbial community in oral cavity^5,10^. In our study, *P. gingivalis* alone showed no effect on expansion or reduction of CD1d^high^CD5^+^ cells in B cells from both immunized and non-immunized mice. However, *P. gingivalis* treatment significantly increased IL-10 secretion in both immunized and non-immunized mice B cells, suggesting this induction was caused by cells other than CD1d^high^CD5^+^ B cells or by increasing the competence of B10 cells in CD1d^high^CD5^+^ subset. Furthermore, *P. gingivalis* treatment significantly diminished the CpG-induced IL-10 production (Figures 4b and 4d) compared with groups without *P. gingivalis* treatment (Figures 4a and 4c) in both innate and adaptive immune responses, suggesting that the IL-10 secretion induced by TLR9 signaling may be inhibited by components of *P. gingivalis.* The mechanism of how *P. gingivalis* induces IL-10 secretion and inhibits TLR9 signaling induced IL-10 secretion in splenocytes B cell needs to be further investigated.

## Conclusions

With CD40L, *P. gingivalis* LPS enhanced IL-10 competency of B10 cells and B10 cell expansion in the absence, but not in the presence of *P. gingivalis*; however, CpG induced the stronger IL-10 competency of B10 cells but inhibited B10 expansion under the same conditions.
